# The impact of time-limited context and feedback methods on epistemic curiosity

**DOI:** 10.3389/fpsyg.2025.1717846

**Published:** 2025-11-17

**Authors:** Zexi Guo, Yibulayin Nadila, Maimaiti Yilizhati

**Affiliations:** Xinjiang Key Laboratory of Mental Development and Learning Science, College of Psychology, Xinjiang Normal University, Ürümqi, China

**Keywords:** epistemic curiosity, time-limited context, feedback, learning motivation, educational psychology

## Abstract

In the school environment, students’ knowledge acquisition typically occurs within a limited-time context that includes external feedback. However, it remains unclear how such contexts influence students’ epistemic curiosity. This study examines the impact of external time limitation and different feedback methods on epistemic curiosity during the learning process through two studies. Study 1 compared epistemic curiosity between limited-time and no-time-limit conditions, finding that participants in the limited-time condition exhibited significantly higher epistemic curiosity. Study 2 investigated the effects of correctness feedback, score feedback, and ranking feedback on epistemic curiosity in a limited-time context, revealing that only correctness feedback significantly enhanced epistemic curiosity. This suggests that external time constraints in the knowledge learning process can stimulate epistemic curiosity, and correctness feedback can sustain this effect.

## 1 Introduction

Epistemic curiosity is an intrinsic motivation for individuals to further explore when facing unknown information (Tang and Salmela-Aro, 2021; Berlyne, 1954). Previous research has found that students with high epistemic curiosity tend to have higher academic achievement (Özsaray and Eren, 2018; Eren and Coskun, 2016) and better learning experience (Litman, 2008). However, the developmental trajectory of epistemic curiosity shows a tendency to decline after students enter formal school education (Engel, 2011; Evans et al., 2023), and the school environment is considered to be detrimental to the development and maintenance of students’ epistemic curiosity (Evans et al., 2023).

In the school environment, time limitation and grade ranking are key factors that influence students’ learning motivation (Seiffge-Krenke, 2008). Once the exam schedule is determined, students are required to complete their knowledge acquisition within a specified timeframe to attain satisfactory results. Current research primarily focuses on how epistemic curiosity promotes students’ learning processes (Schumacher et al., 2025; Ma et al., 2025), but lacks in exploration of its influencing factors in the school environment. This limits our understanding of how the school environment shapes students’ epistemic curiosity and complicates the development of educational improvement strategies. Therefore, this study employs an empirical research approach to examine how the time-limited context for knowledge learning and teaching feedback in the school environment impacts students’ epistemic curiosity, aiming to provide references for future educational practice.

### 1.1 Time-limited context and epistemic curiosity

Examination pressure is a pivotal factor within the school environment that impacts students’ curiosity (Jirout et al., 2024; Engel, 2011). In particular, the limited time for knowledge acquisition caused by examination schedules is often cited as a major cause of the decline in students’ curiosity during the learning process (Engel, 2011; Evans et al., 2023).

**Figure 1:**
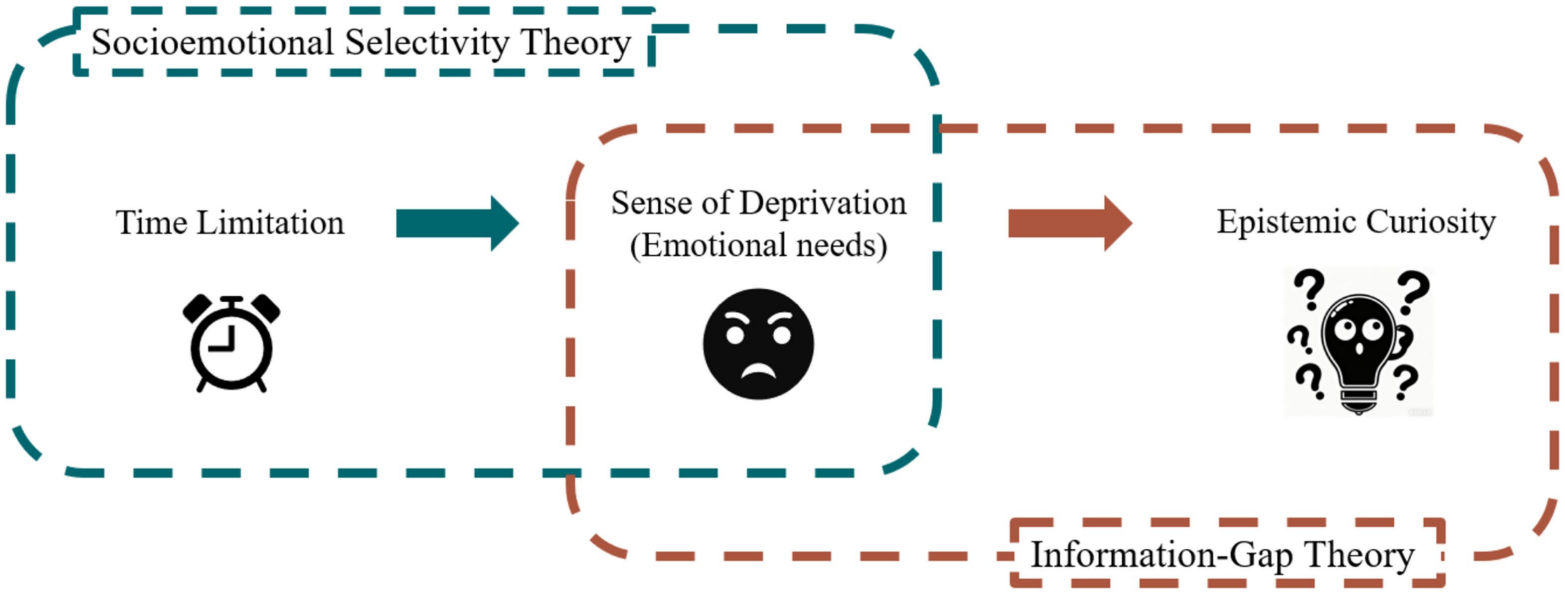
The conceptual model of the research.

However, in daily life, time limitation may also enhance curiosity rather than suppress it. For instance, books borrowed from the library seem to attract readers more than those that have been purchased. The sole distinction between the two lies in the presence or absence of an external time limitation on reading. The socioemotional selectivity theory (SST) suggests that when individuals perceive time as limited, they are more likely to prioritize their emotional needs (Carstensen, 2006). In the preceding context, it is not the book itself that attracts individuals, but rather the unknown information it contains. This thirst for knowledge, sparked by the unknown information, is termed epistemic curiosity (Keller et al., 2024; Berlyne, 1954). According to the Information-Gap Theory of curiosity, epistemic curiosity is closely linked to negative emotional experiences arising from unknown information (Huang et al., 2021; Loewenstein, 1994). Specifically, when individuals perceive a discrepancy between what they know and what they wish to know, they experience an aversive emotional state of deprivation, which motivates them to seek information to reduce this discomfort, thus demonstrating a higher level of epistemic curiosity (Litman, 2008; Huang et al., 2021). Empirical findings support this theoretical account. Van Lieshout et al. (2021) found that participants’ curiosity increased with greater uncertainty about unknown information, even as their subjective well-being decreased. Complementing these behavioral results, neurocognitive evidence also indicates that curiosity involves dopaminergic systems associated with emotional arousal (Gruber and Ranganath, 2019). Building on these findings, it can be inferred that time limitation amplifies the sense of deprivation from unknown information through increased sensitivity to emotional needs, a mechanism explained by SST; this sense of deprivation constitutes a key negative emotional experience in the Information-Gap Theory and serves as the core driver of epistemic curiosity (Figure 1).

Therefore, under time-limited contexts, unknown information may become more appealing. In other words, individuals may exhibit higher epistemic curiosity during the knowledge acquisition process in time limitation situations compared to those without constraints.

### 1.2 The impact of feedback methods on epistemic curiosity in time-limited contexts

The Agenda-Based Regulation model suggests that individuals prioritize high-reward tasks during learning (Ariel et al., 2009). In results-oriented school settings, this manifests as students allocating most of their limited study time to exam-related content (Khoshhal et al., 2017). Here, external factors like high grades dominate their learning motivation, while internal motivation like curiosity may often be neglected. According to the causality orientations theory, external control that undermines basic psychological needs (e.g., autonomy) can weaken internal motivation (Zhao et al., 2023). Epistemic curiosity, as an internal driver for knowledge acquisition (Tang and Salmela-Aro, 2021; Berlyne, 1954), may be affected by external feedback, especially in time-limited contexts.

Exam scores can be regarded as a form of evaluative feedback provided by teachers, reflecting students’ mastery of knowledge (Sirianansopa, 2024; Hattie and Timperley, 2007). Based on the way the information is conveyed, feedback can be categorized into informational feedback and controlling feedback (Wisniewski et al., 2020; Ryan, 1982). Informational feedback (e.g., “You did really well.”) simply describes the outcome of behavior, signaling that the recipient can control their actions. It helps maintain and enhance autonomy, thereby sustaining or reinforcing internal motivation (Ryan, 1982). In contrast, controlling feedback (e.g., “You did very well, just as you should.”) emphasizes expected standards, highlighting external control. This can increase the sense of being controlled, reducing autonomy and undermining internal motivation (Ryan, 1982). Zhou (1998) found that informational feedback enhances intrinsic motivation more effectively than controlling feedback. Epistemic curiosity, as a dominant factor of internal learning motivation (Murayama, 2022; Litman, 2008), may be affected by different types of feedback under time-limited conditions.

In teaching practice, common feedback includes exam scores and rankings (Kajitani et al., 2020), as well as simple correctness judgments of students’ answers. From a feedback perspective, correctness feedback merely informs students of their performance and serves as informational feedback, which can enhance students’ sense of autonomy. In contrast, ranking feedback conveys external demands and is considered controlling feedback, which may weaken students’ experience of autonomy. According to Self-Determination Theory (Deci and Ryan, 2000), the degree to which individuals experience autonomy in their actions directly influences their intrinsic motivation. When feedback is perceived as autonomy-supportive, such as correctness feedback, it nurtures students’ intrinsic motivation, which is reflected in higher levels of epistemic curiosity. Conversely, when feedback is perceived as controlling, such as ranking feedback, it shifts motivation toward compliance with external standards, thereby reducing students’ intrinsic motivation and resulting in lower levels of epistemic curiosity. Moreover, in the current results-oriented school teaching environment (Jirout et al., 2024), scores have shifted from being merely an evaluation tool to becoming dominant performance targets that drive instructional decisions, sometimes at the expense of broader learning goals (Khan et al., 2025; Huang and Zeng, 2025). This transformation endows them with an external control aspect, making them more akin to controlling feedback, which may diminish students’ epistemic curiosity.

Overall, in the school teaching environment, teacher feedback typically follows time-limited knowledge learning. How different types of teacher feedback affect students’ epistemic curiosity during the time-limited knowledge acquisition process still requires further exploration.

### 1.3 Purpose of the present study

Students’ knowledge acquisition predominantly takes place within the campus environment, which is characterized by limited learning time and subsequent feedback. Examining how students’ epistemic curiosity evolves in this context can provide valuable insights for teaching improvement plans aimed at enhancing their curiosity. Accordingly, this study addresses the following research questions: (1) Does a time-limited situation enhance epistemic curiosity during students’ knowledge learning process? (2) How does epistemic curiosity change in a time-limited situation under three types of feedback: correctness, score, and ranking?

## 2 Study1

### 2.1 Method

#### 2.1.1 Participants

Using the G*Power 3.1 software and referring to the study by Hsee and Ruan (2016), the effect size was set at *f* = 0.8. The results indicated that a total of 42 participants were required for the study (1 − *β* = 0.8, *α* = 0.05). For Study 1, 108 Asian middle school students were recruited through public solicitation, including 52 males, with an average age of 15.15 ± 1.56. Before the experiment, informed consent was obtained from the participants, who were required to have normal reading and comprehension abilities, as well as normal or corrected-to-normal vision.

#### 2.1.2 Procedure

Considering that in actual students learning scenarios, the experience of time limitation arises not from minute-by-minute countdowns, but rather from evaluating the number of unfinished tasks and available learning opportunities, this study enhanced ecological validity by adapting the design of Wang et al. (2021). A time-limited learning situation was created by restricting the number of task rounds.

Participants were informed that they were taking part in a knowledge popularization activity. Forty question-and-answer items were prepared as learning materials, drawn randomly from the pool compiled by Fastrich et al. (2018). The entire learning phase was divided into 10 rounds. In each round, participants could freely choose to learn 1, 2, or 3 items. Importantly, even if a participant selected the maximum number of items (3) in every round, they would complete only 30 items across the 10 rounds, leaving at least 10 items unlearned. Thus, there were always items that remained unknown, which served as the target for curiosity assessment.

Before the learning phase began, participants were presented with a sample item to familiarize themselves with the task format. Following the method of Gruber et al. (2014), participants then rated their epistemic curiosity about the unlearned items on a 7-point scale (1 = not at all, 7 = very much), with higher scores indicating greater curiosity. After the 10-round learning phase, participants again reported their epistemic curiosity, this time specifically regarding the items that remained unlearned in the final round.

To examine the effectiveness of the manipulation, participants were also asked: “To what extent did you experience the feeling of time limitation in the learning process during the last round?” (1 = not at all, 5 = completely). Participants in the time-limited group were informed of the total number of rounds before the experiment, whereas those in the control group were not.

**Figure 2:**
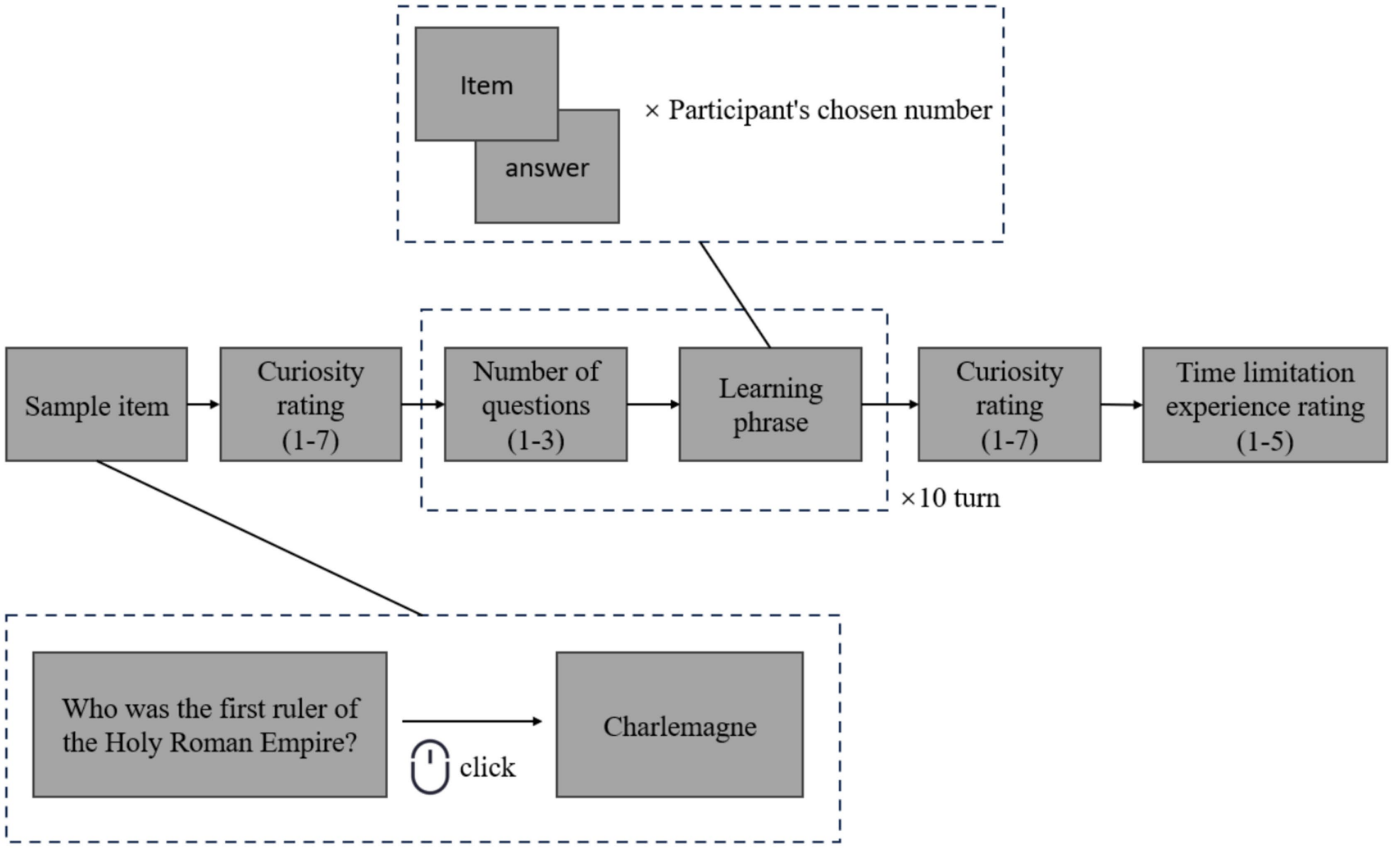
Study 1 procedure diagram.

The study procedure is illustrated in Figure 2. This study was approved by the Research Committee of the Department of Psychology at Xinjiang Normal University (approval number 2025009), and all participants provided informed consent prior to participation.

### 2.2 Result

Data analysis was conducted using R 4.4.2. A mixed-design ANOVA was performed with epistemic curiosity as the dependent variable, including a between-subjects factor of time limitation (present vs. absent) and a within-subjects factor of measurement occasion (pretest vs. posttest). Prior to conducting the mixed-design ANOVA, several assumptions were examined. The dependent variable (epistemic curiosity) was treated as continuous, and the independence of observations was ensured by the experimental design. Box’s M test was used to assess the homogeneity of covariance matrices, and Levene’s test was applied to examine the homogeneity of variances. The results indicated that both assumptions were met (Box’s M, *p* > 0.05; Levene’s test, *p*s > 0.05). Given the sufficiently large sample size in each group (*n* > 30), the parametric analyses can be considered robust to potential deviations from normality (Blanca Mena et al., 2017). Additionally, an independent samples *t*-test was conducted to examine the effectiveness of the manipulation of the time limitation condition.

#### 2.2.1 Experimental manipulation assessment

The independent samples *t*-test revealed that participants in the time limitation present conditions reported significantly higher levels of subjective time-limited experiences compared to those in the no time limitation conditions [*t* = −12.01, *p* < 0.05, *Cohen’s d* = −2.311, 95% *CI* (−2.83, −1.76), large effect]. This finding demonstrates that the manipulation of the time limitation in this study was successful.

#### 2.2.2 Descriptive analysis

In Study 1, participants were divided into two groups, each consisting of 54 individuals. Table 1 presents the pretest and posttest levels of participants’ curiosity about unknown information under different experimental conditions.

**Table 1:** Epistemic curiosity ratings under different conditions (M ± SD).

Experimental conditions	*n*	Pretest	Posttest
Time limitation present	54	5.19 ± 1.10	6.19 ± 0.83
Time limitation absent	54	5.03 ± 1.49	5.26 ± 1.57

#### 2.2.3 Major analysis

**Figure 3:**
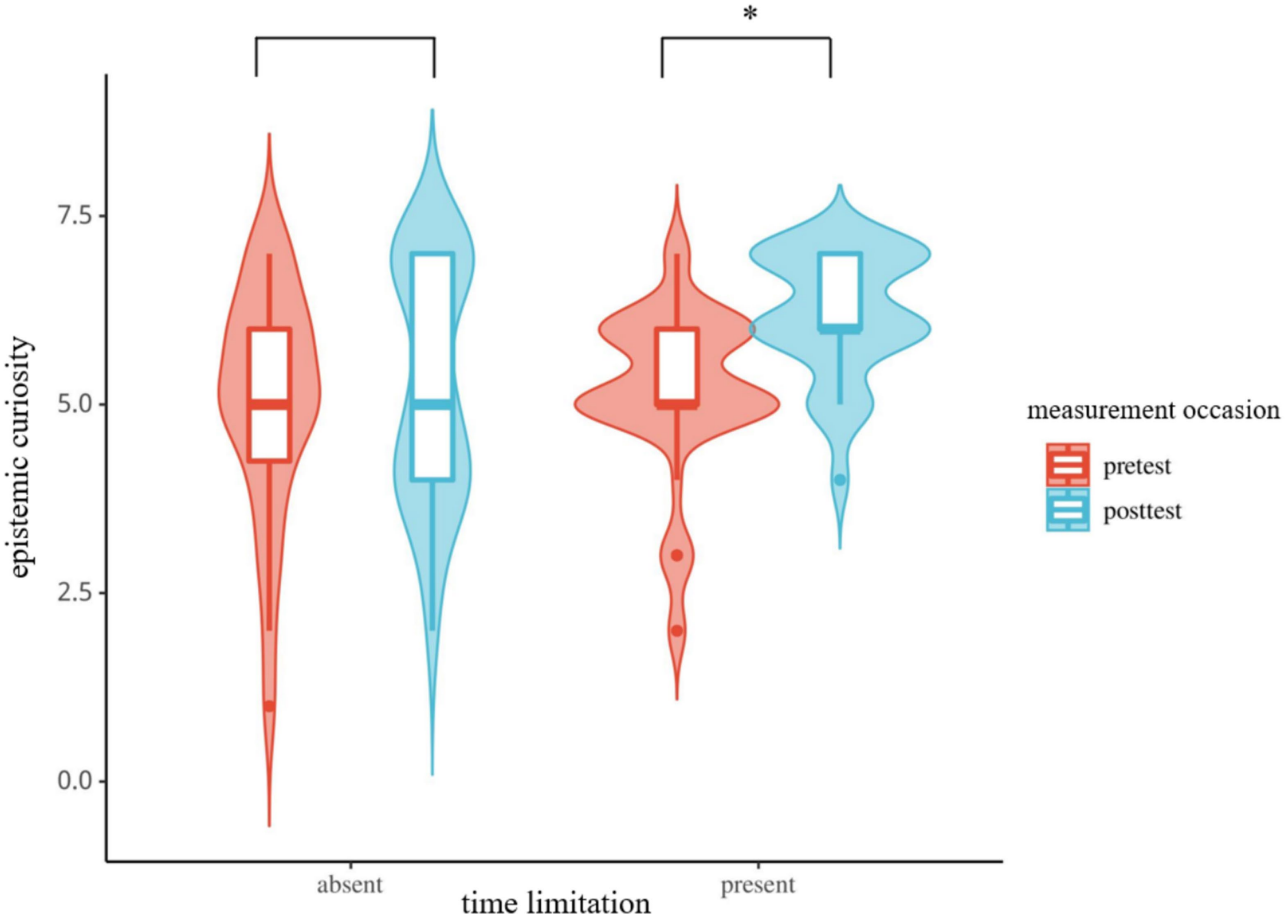
Epistemic curiosity ratings under different conditions (*p < 0.05).

The results of ANOVA indicated significant main effects of time limitation and measurement occasion, as well as a significant interaction between them (as shown in Figure 3). Specifically, the main effect of time limitation was significant, *F* (1, 53) = 26.69, *p* < 0.05, *η*^2^ = 0.34, 95% *CI* (0.17, 0.48), representing a large effect. Students exhibiting higher epistemic curiosity under time limitation present conditions compared to no time limitation conditions. The main effect of measurement time was also significant, *F* (1, 53) = 8.46, *p* < 0.05, *η*^2^ = 0.14, 95% *CI* (0.03, 0.28), representing a large effect, with higher epistemic curiosity at the posttest compared to the pretest. The interaction between time limitation and measurement time was significant, *F* (1, 53) = 4.69, *p* < 0.05, *η*^2^ = 0.08, 95% *CI* (0.00, 0.20), representing a medium effect. Simple effect analysis revealed no significant difference in epistemic curiosity between time limitation present conditions at the pretest [*p* = 0.45, *η*^2^ = 0.01, 95% *CI* (0.00, 0.05), small effect], but a significant difference at the posttest, with higher curiosity under time limitation conditions [*p* < 0.05, *η*^2^ = 0.25, 95% *CI* (0.12, 0.40), large effect].

#### 2.2.4 Discussion

Study 1 created a time-limited learning situation by informing participants of the total number of learning rounds and examined its impact on individuals’ epistemic curiosity. The results supported the hypothesis that under time-limited conditions, unknown information would trigger a higher level of epistemic curiosity. According to the socioemotional selectivity theory, external time limitation enhance individuals’ focus on their emotional needs (Xing et al., 2019; Carstensen, 2006). Thus, in this study, epistemic curiosity—an emotional need arising from facing unknown information—is further enhanced under time-limited learning conditions.

However, while Study 1 confirmed that time limitation promotes epistemic curiosity among students in the learning process, in school environments, time-limited learning situations are typically followed by subsequent teaching feedback. To further explore the impact of the school learning environment on students’ epistemic curiosity, Study 2 will examine how different feedback methods influence the effect of time-limited contexts on enhancing individuals’ epistemic curiosity.

## 3 Study 2

### 3.1 Method

#### 3.1.1 Participants

Using the G*Power 3.1 software and referring to the study by Hsee and Ruan (2016), the effect size was set at *f* = 0.8. The results indicated that a total of 21 participants were required for the study (1 − *β* = 0.8, *α* = 0.05). For Study 2, 120 Asian middle school students were recruited through public solicitation, including 68 males, with an average age of 15.15 ± 1.56 years. Before the experiment, informed consent was obtained from the participants, who were required to have normal reading and comprehension abilities, as well as normal or corrected-to-normal vision.

#### 3.1.2 Produce

Similar to Study 1, participants were informed that they were taking part in a knowledge popularization activity. Forty question-and-answer items were prepared as the learning materials, randomly selected from the item pool compiled by Fastrich et al. (2018). The entire learning process was divided into 10 rounds, in each of which participants could freely choose to learn 1, 2, or 3 items. Importantly, even if a participant selected the maximum number of items (3) in every round, they would complete only 30 items across the 10 rounds, leaving at least 10 items unlearned. Thus, there were always items that remained unknown, which served as the target for curiosity assessment.

Before the learning phase, participants were first presented with a sample item to familiarize themselves with the task format. Next, they were informed about the type of feedback they would receive after a supposed test phase and were randomly assigned to one of three feedback conditions. In the correctness feedback condition, participants were told that they would receive information indicating whether each response was correct or incorrect. In the score feedback condition, they were informed that their performance would be summarized as a total score based on the number of correct answers. In the ranking feedback condition, participants were told that they would be given their relative performance compared with others, expressed as a ranking position. To enhance ecological validity and induce a realistic sense of feedback anticipation, participants were informed that a knowledge test would follow the learning phase. However, this test was not actually administered. Instead, feedback was generated according to pre-designed scripts to ensure the credibility of the manipulation while avoiding potential confounds related to actual performance assessment.

Epistemic curiosity was measured at two points. Before the learning phase, participants rated their curiosity about the unlearned items on a 7-point scale (1 = not at all, 7 = very much). After the 10-round learning phase, they again reported their curiosity, specifically regarding the items that remained unlearned in the final round.

**Figure 4:**
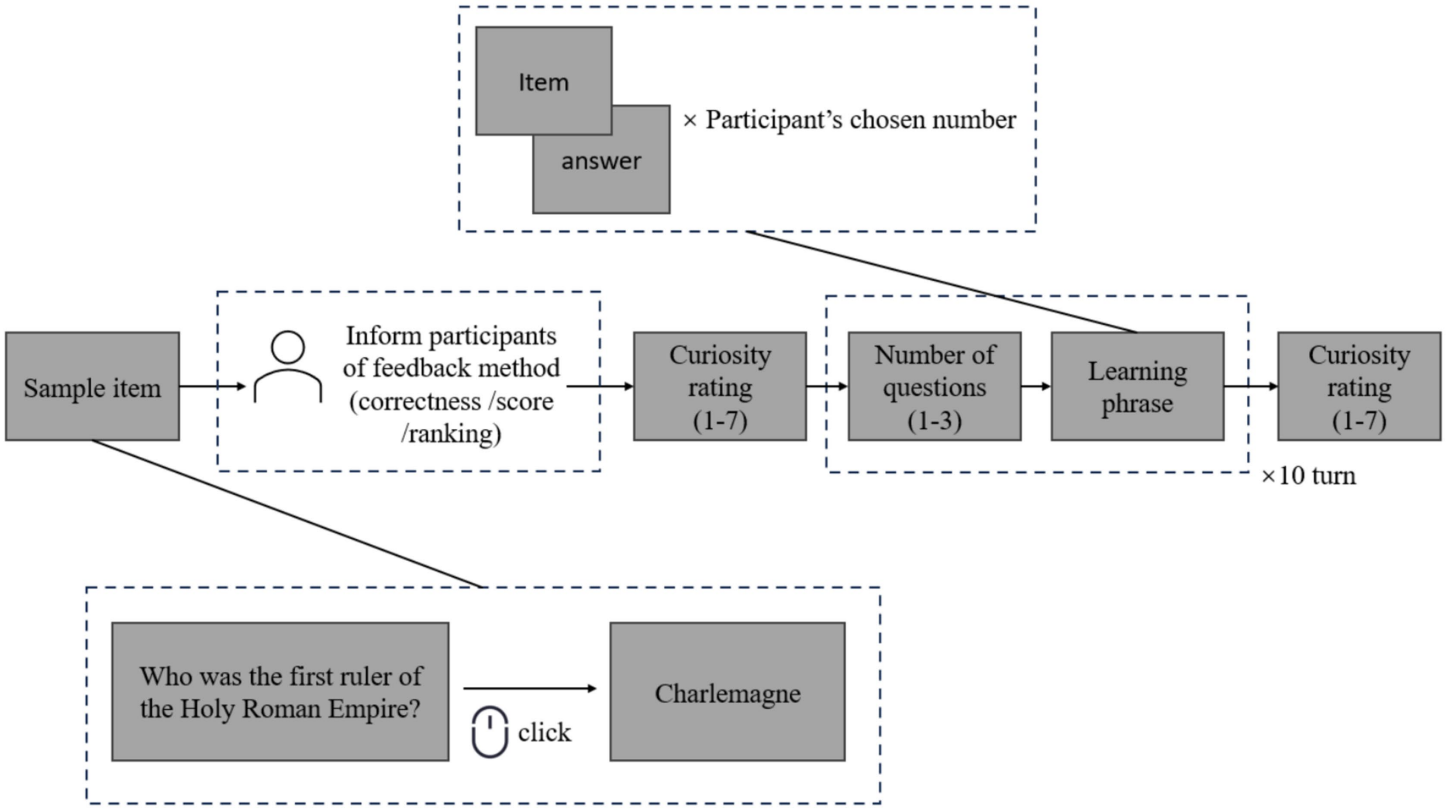
Study 2 procedure diagram.

The study procedure is illustrated in Figure 4. This study was approved by the Research Committee of the Department of Psychology at Xinjiang Normal University (approval number 2025009), and all participants provided informed consent prior to participation.

### 3.2 Result

Data analysis was conducted using R 4.4.2. A mixed-design ANOVA was performed with epistemic curiosity as the dependent variable, including a between-subjects factor of feedback (correctness vs. score vs. ranking) and a within-subjects factor of measurement occasion (pretest vs. posttest). Prior to conducting the mixed-design ANOVA, several assumptions were examined. The dependent variable (epistemic curiosity) was treated as continuous, and the independence of observations was ensured by the experimental design. Box’s M test was used to assess the homogeneity of covariance matrices, and Levene’s test was applied to examine the homogeneity of variances. The results indicated that both assumptions were met (Box’s M, *p* > 0.05; Levene’s test, *p*s > 0.05). Given the sufficiently large sample size in each group (*n* > 30), the parametric analyses can be considered robust to potential deviations from normality (Blanca Mena et al., 2017).

#### 3.2.1 Descriptive analysis

In Study 2, participants were divided into three groups, each consisting of 40 individuals. Table 2 presents the pretest and posttest levels of participants’ curiosity about unknown information under different experimental conditions.

**Table 2:** Epistemic curiosity ratings under different feedback method (M ± SD).

Feedback method	*n*	Pretest	Posttest
Correctness	40	5.15 ± 1.11	6.1 ± 0.89
Score	40	4.95 ± 1.43	5.25 ± 1.22
Ranking	40	4.95 ± 1.63	4.75 ± 1.58

#### 3.2.2 Major analysis

**Figure 5:**
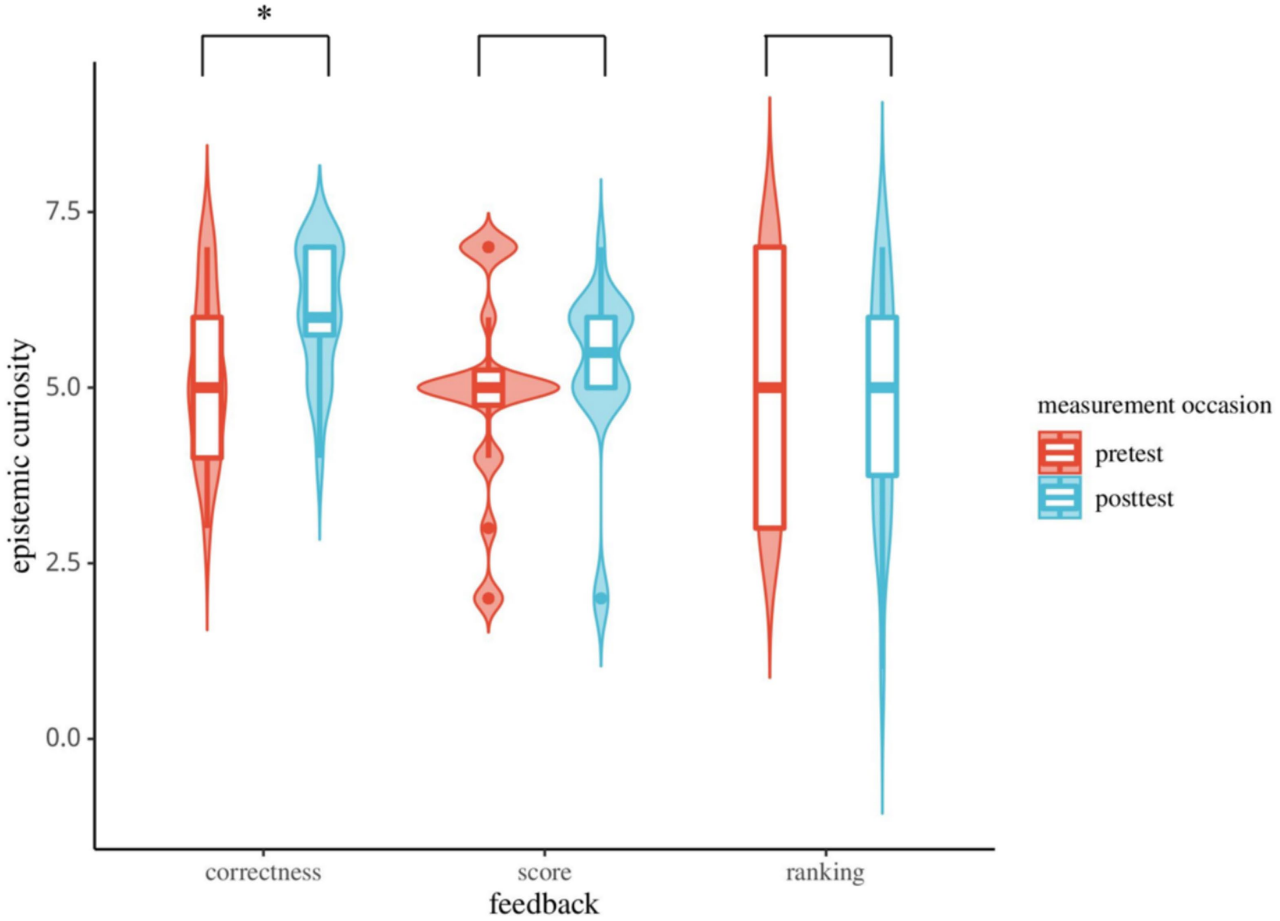
Epistemic curiosity ratings under different feedback method (*p < 0.05).

The results of ANOVA revealed significant main effects of feedback method and measurement occasion, as well as a significant interaction between them (as shown in Figure 5). The results showed that the main effect of feedback method was significant, *F* (2, 78) = 4.97, *p* < 0.05, *η*^2^ = 0.11, 95% *CI* (0.02, 0.24), representing a medium effect, indicating substantial differences in students’ epistemic curiosity across various feedback conditions. The main effect of measurement time was also significant, *F* (2, 78) = 4.75, *p* < 0.05, *η*^2^ = 0.11, 95% *CI* (0.02, 0.23) also a medium effect, showing that students’ epistemic curiosity was notably higher at the posttest compared to the pretest.

The interaction between feedback method and measurement occasion was significant, *F* (2, 78) = 7.20, *p* < 0.05, *η*^2^ = 0.16, 95% *CI* (0.04, 0.30), representing a large effect. Simple effect analysis revealed that in the correctness condition, students’ epistemic curiosity was significantly higher at the posttest time point than at the pretest [*p* < 0.05, *η*^2^ = 0.42, 95% *CI* (0.23, 0.55), large effect]. In contrast, in the score feedback condition, there was no significant difference in epistemic curiosity between the pretest and posttest [*p* = 0.11, *η*^2^ = 0.06, 95% *CI* (0.00, 0.15), medium effect]. Similarly, in the ranking feedback condition, no significant difference was observed between the pretest and posttest epistemic curiosity levels [*p* = 0.54, *η*^2^ = 0.01, 95% *CI* (0.00, 0.05), small effect].

### 3.3 Discussion

Study 2 combined the limited-time condition with different feedback methods, allowing students to learn knowledge under the expectation of specific instructional feedback. This study aimed to examine the impact of various feedback methods on the epistemic curiosity of middle school students in a limited-time situation. The results indicated that the limited-time condition significantly enhanced epistemic curiosity when combined with correctness feedback. However, in the conditions involving test scores and ranking feedback, the limited-time situation did not promote epistemic curiosity. Additionally, these two feedback methods did not show significant differences in their impact on individual epistemic curiosity.

## 4 Discussion

This study, starting from the characteristics of the school environment, examines the impact of the inherent time-limited context of school knowledge learning on individual epistemic curiosity and how this impact varies under different teaching feedback conditions. The results showed that the time-limited context can enhance epistemic curiosity during the learning process, and feedback on correctness can sustain this effect.

### 4.1 Higher epistemic curiosity in time-limited context

According to the socioemotional selectivity theory, changes in time perception can lead to shifts in individual motivation (Xing et al., 2019). When time is perceived as unlimited, individuals tend to focus more on long-term goals; however, when time is perceived as limited, individuals pay more attention to their emotional needs (Xing et al., 2019).

In previous research on curiosity, curiosity has often been regarded as an internal motivation to eliminate subjective discomfort. According to the information gap theory, the presence of unknown information can trigger a “deprivation” feeling in individuals, activating negative emotional experiences. The emergence of curiosity is related to this negative experience. van Lieshout et al. (2021) supported this view in their study, in which participants engaged in a lottery task where they could see the probability of winning. The researchers manipulated the winning probability and found that when the winning probability was low, participants’ subjective pleasure was lower, but their curiosity about the lottery results was higher. Beyond this affective explanation, Cai et al. (2018) found that time-limited situations elicit a heightened sense of task urgency and perceived information scarcity, and this perception of scarcity serves as an important psychological mechanism that stimulates curiosity. In other words, when time is constrained, individuals may form a mental representation that the opportunity to obtain information is about to be lost, which intensifies their attention to and desire for that information. This interpretation aligns with the scarcity effect theory, which posits that scarcity increases the subjective value and attractiveness of an object or resource (Shah et al., 2012). The elevated subjective value may, in turn, evoke stronger feelings of deprivation and other negative emotional experiences, thereby promoting higher levels of epistemic curiosity. In summary, the increase in epistemic curiosity among middle school students in a time-limited context in this study can be understood as the time-limited context enhancing the negative emotional experience brought about by unknown information, thereby triggering a higher level of epistemic curiosity in middle school students.

It is worth noting that in the pretest phase of Study 1, participants in the time-limited group had already been informed of the total number of learning rounds. However, at this point, there was no significant difference in epistemic curiosity between the two groups. The difference only emerged in the posttest phase after the learning process. One possible reason is that before the learning phase began, participants had not yet truly experienced the external time constraints. Another possibility is that the impact of the time-limited context on emotional needs may only manifest after these needs have already developed. Overall, middle school students’ expectation of limited learning time does not directly enhance their epistemic curiosity levels.

### 4.2 Correctness feedback enhances epistemic curiosity in time-limited contexts

Curiosity can drive learning behavior, reflecting its intrinsic motivational nature (Tang and Salmela-Aro, 2021; Berlyne, 1954). Intrinsic motivation is largely influenced by individuals’ experiences of autonomy (Conesa et al., 2023). As previously mentioned, correctness feedback is informational and can maintain middle school students’ sense of control and autonomy during knowledge acquisition. Therefore, it sustains the promoting effect of the time-limited context on their epistemic curiosity. In contrast, under the ranking feedback condition, the external control of “achieving a good rank” is always present, driving part of the learning behavior through external motivation. According to cognitive evaluation theory, this external control can weaken intrinsic motivation by undermining autonomy (Al-Hoorie et al., 2025; Ryan et al., 1983). Thus, under the ranking feedback condition, the promoting effect of the time-limited context on epistemic curiosity disappeared. The findings align with prior research, such as the study conducted by Conesa et al. (2023), which demonstrated that students exhibit higher levels of motivation when exposed to a less controlling teaching style.

Under the score feedback condition, consistent with the ranking feedback condition, the promoting effect of the time-limited context on epistemic curiosity disappeared. This result indicates that test scores, like grade rankings, possess a similar attribute of external control. Initially, test scores served merely as a quantitative measure of correctness and an objective basis for assessing knowledge acquisition. However, in the current results-oriented educational environment (Jirout et al., 2024), scores have evolved from being just an evaluation tool to becoming a primary teaching goal. When students consider test scores during learning, their intrinsic motivation to learn is undermined. This is evident in our study, where the time-limited context no longer enhances epistemic curiosity.

### 4.3 Limitation

This study highlights that autonomy plays a central role in the changes of epistemic curiosity among middle school students. However, to prevent participants from guessing the purpose of the study, the changes in students’ autonomy during the learning process were not directly measured. Future research should further examine the role of students’ autonomy experience in the emergence of epistemic curiosity.

Another limitation concerns potential expectancy or demand effects introduced by the feedback manipulation. As participants anticipated receiving feedback (even though no actual test was administered), their curiosity ratings might have been influenced by perceived experimenter expectations or self-presentation concerns. Future studies could adopt a double-blind design or include measures of social desirability to control for these effects.

### 4.4 Implications for practice

Acquiring knowledge is a crucial task for students, and assisting them in this endeavor is the primary responsibility of teachers. This study examines the changes in middle school students’ epistemic curiosity regarding new knowledge, starting from the inherent time constraints and feedback mechanisms within the school teaching environment. The results indicate that under time-limited conditions, middle school students’ epistemic curiosity towards new knowledge is enhanced. Providing correctness feedback can sustain this enhanced effect. In contrast, feedback based on scores and rankings tends to eliminate this positive effect. The findings hold significance for subsequent teaching practices.

Firstly, this study delves deeper into the influence of the school teaching environment on students’ epistemic curiosity (Evans et al., 2023). It establishes that a time-limited context can significantly boost epistemic curiosity during the process of knowledge acquisition. Secondly, the study reveals that correctness feedback can effectively sustain the boost in epistemic curiosity triggered by time-limited contexts. This suggests that integrating correctness feedback with time limitation in teaching can maximize the enhancement of students’ epistemic curiosity.

To put this into practice, examinations, a commonly used assessment tool in school education, also serve as an effective means of creating time-limited learning contexts. Teachers can include non-scored bonus questions in exams. These questions encourage students to apply their existing knowledge without the pressure of grades, fostering genuine curiosity and the intrinsic joy of learning. Moreover, given the current results-oriented teaching environment (Jirout et al., 2024), exams often evoke a strong sense of external control. It is essential to move beyond the singular reliance on traditional paper-and-pencil tests for assessing learning outcomes. Considering the typical classroom structure in schools, innovative classroom formats such as student presentations and student-led teaching sessions offer effective alternatives.

## 5 Discussion

The limited-time context can enhance epistemic curiosity. However, this effect is compromised under conditions involving scores and ranking feedback, while it remains intact under the correctness feedback condition.

## Data Availability

The datasets presented in this study can be found in online repositories. The names of the repository/repositories and accession number(s) can be found below: https://osf.io/84psx/.

## References

[ref1] Al-Hoorie A. H. Oga-Baldwin W. Q. Hiver P. Vitta J. P. (2025). Self-determination mini-theories in second language learning: a systematic review of three decades of research. Lang. Teach. Res. 29, 1603–1638. doi: 10.1177/13621688221102686

[ref2] Ariel R. Dunlosky J. Bailey H. (2009). Agenda-based regulation of study-time allocation: when agendas override item-based monitoring. J. Exp. Psychol. Gen. 138, 432–447. doi: 10.1037/a0015928, PMID: 19653800

[ref3] Berlyne D. E. (1954). A theory of human curiosity. Br. J. Psychol. 45, 180–191. doi: 10.1111/j.2044-8295.1954.tb01243.x, PMID: 13190171

[ref4] Blanca Mena M. J. Alarcón Postigo R. Arnau Gras J. Bono Cabré R. Bendayan R. (2017). Non-normal data: Is ANOVA still a valid option? Psicothema 4, 552–557. doi: 10.7334/psicothema2016.383, PMID: 29048317

[ref9001] Cai Z. G. Wang R. Shen M. Speekenbrink M. (2018). Cross-dimensional magnitude interactions arise from memory interference. Cogn. Psychol. 106, 21–42. doi: 10.1016/j.cogpsych.2018.08.001 30165241

[ref5] Carstensen L. L. (2006). The influence of a sense of time on human development. Science 312, 1913–1915. doi: 10.1126/science.1127488, PMID: 16809530 PMC2790864

[ref6] Conesa P. J. Dunabeitia J. A. Onandia-Hinchado I. González-Cutre D. (2023). Satisfying students' psychological needs in the classroom: benefits of an online intervention to help primary school teachers during a pandemic academic year. Teach. Teach. Educ. 133:104281. doi: 10.1016/j.tate.2023.104281

[ref7] Deci E. L. Ryan R. M. (2000). The" what" and" why" of goal pursuits: human needs and the self-determination of behavior. Psychol. Inq. 11, 227–268. doi: 10.1207/s15327965pli1104_01

[ref8] Engel S. (2011). Children's need to know: curiosity in schools. Harv. Educ. Rev. 81, 625–645. doi: 10.17763/haer.81.4.h054131316473115

[ref9] Eren A. Coskun H. (2016). Students’ level of boredom, boredom coping strategies, epistemic curiosity, and graded performance. J. Educ. Res. 109, 574–588. doi: 10.1080/00220671.2014.999364

[ref10] Evans N. S. Burke R. Vitiello V. Zumbrunn S. Jirout J. J. (2023). Curiosity in classrooms: an examination of curiosity promotion and suppression in preschool math and science classrooms. Think. Skills Creat. 49:101333. doi: 10.1016/j.tsc.2023.101333

[ref11] Fastrich G. M. Kerr T. Castel A. D. Murayama K. (2018). The role of interest in memory for trivia questions: an investigation with a large-scale database. Motiv. Sci. 4, 227–250. doi: 10.1037/mot0000087, PMID: 30221181 PMC6133257

[ref12] Gruber M. J. Gelman B. D. Ranganath C. (2014). States of curiosity modulate hippocampus-dependent learning via the dopaminergic circuit. Neuron 84, 486–496. doi: 10.1016/j.neuron.2014.08.060, PMID: 25284006 PMC4252494

[ref13] Gruber M. J. Ranganath C. (2019). How curiosity enhances hippocampus-dependent memory: the prediction, appraisal, curiosity, and exploration (PACE) framework. Trends Cogn. Sci. 23, 1014–1025. doi: 10.1016/j.tics.2019.10.003, PMID: 31706791 PMC6891259

[ref14] Hattie J. Timperley H. (2007). The power of feedback. Rev. Educ. Res. 77, 81–112. doi: 10.3102/003465430298487

[ref15] Hsee C. K. Ruan B. (2016). The Pandora effect: the power and peril of curiosity. Psychol. Sci. 27, 659–666. doi: 10.1177/0956797616631733, PMID: 27000178

[ref16] Huang Q. Cao S. Zhou S. Punia D. Zhu X. Luo Y. . (2021). How anxiety predicts interpersonal curiosity during the COVID-19 pandemic: the mediation effect of interpersonal distancing and autistic tendency. Personal. Individ. Differ. 180:110973. doi: 10.1016/j.paid.2021.110973, PMID: 34629580 PMC8487302

[ref17] Huang L. Zeng J. (2025). Sustainability of washback effects: a longitudinal study of China’s twice-yearly NMET reform. Lang. Test. Asia 15:19. doi: 10.1186/s40468-025-00358-9

[ref18] Jirout J. J. Evans N. S. Son L. K. (2024). Curiosity in children across ages and contexts. Nat. Rev. Psychol. 3, 622–635. doi: 10.1038/s44159-024-00346-5

[ref19] Kajitani S. Morimoto K. Suzuki S. (2020). Information feedback in relative grading: evidence from a field experiment. PLoS One 15:e0231548. doi: 10.1371/journal.pone.0231548, PMID: 32311001 PMC7170246

[ref20] Keller N. E. Salvi C. Leiker E. K. Gruber M. J. Dunsmoor J. E. (2024). States of epistemic curiosity interfere with memory for incidental scholastic facts. NPJ Sci. Learn. 9:22. doi: 10.1038/s41539-024-00234-w, PMID: 38499583 PMC10948872

[ref21] Khan A. Hassan N. Cheng L. (2025). Investigating the contextual factors mediating washback effects of a learning-oriented English language assessment in Malaysia. Lang. Test. Asia 15:20. doi: 10.1186/s40468-025-00359-8

[ref22] Khoshhal K. I. Khairy G. A. Guraya S. Y. Guraya S. S. (2017). Exam anxiety in the undergraduate medical students of Taibah University. Med. Teach. 39, S22–S26. doi: 10.1080/0142159x.2016.1254749, PMID: 28103727

[ref23] Litman J. A. (2008). Interest and deprivation factors of epistemic curiosity. Pers. Individ. Differ. 44, 1585–1595. doi: 10.1016/j.paid.2008.01.014

[ref24] Loewenstein G. (1994). The psychology of curiosity: a review and reinterpretation. Psychol. Bull. 116, 75–98. doi: 10.1037/0033-2909.116.1.75

[ref25] Ma S. Jin X. Li X. Dong H. Dong X. Tang B. (2025). How epistemic curiosity influences digital literacy: evidence from international students in China. Behav. Sci. 15:286. doi: 10.3390/bs15030286, PMID: 40150181 PMC11939729

[ref26] Murayama K. (2022). A reward-learning framework of knowledge acquisition: an integrated account of curiosity, interest, and intrinsic–extrinsic rewards. Psychol. Rev. 129, 175–198. doi: 10.1037/rev0000349, PMID: 35099213

[ref27] Özsaray A. E. Eren A. (2018). Achievement emotions, epistemic curiosity, and graded performance of undergraduate students in English preparatory classes. Uluslararası Egitim Programları Dergisi 8, 39–58. doi: 10.31704/ijocis.2018.003

[ref28] Ryan R. M. (1982). Control and information in the intrapersonal sphere: an extension of cognitive evaluation theory. J. Pers. Soc. Psychol. 43, 450–461. doi: 10.1037/0022-3514.43.3.450

[ref29] Ryan R. M. Mims V. Koestner R. (1983). Relation of reward contingency and interpersonal context to intrinsic motivation: a review and test using cognitive evaluation theory. J. Pers. Soc. Psychol. 45, 736–750. doi: 10.1037/0022-3514.45.4.736

[ref30] Schumacher A. Kammerer Y. Scharinger C. Gottschling S. Hübner N. Tibus M. . (2025). How do intellectually curious and interested people learn and attain knowledge? A focus on behavioral traces of information seeking. Eur. J. Personal. 08902070241309124. [online ahead of print]. doi: 10.1177/08902070241309124

[ref31] Seiffge-Krenke I. (2008). Some consequences of having too little. Prax. Kinderpsychol. Kinderpsychiatr. 57, 3–19. doi: 10.13109/prkk.2008.57.1.3, PMID: 18361182

[ref9002] Shah A. K. Mullainathan S. Shafir E. (2012). Some consequences of having too little. Sci. 338, 682–685. doi: 10.1126/science.1222426 23118192

[ref32] Sirianansopa K. (2024). Evaluating students’ learning achievements using the formative assessment technique: a retrospective study. BMC Med. Educ. 24:1373. doi: 10.1186/s12909-024-06347-5, PMID: 39593083 PMC11590208

[ref33] Tang X. Salmela-Aro K. (2021). The prospective role of epistemic curiosity in national standardized test performance. Learn. Individ. Differ. 88:102008. doi: 10.1016/j.lindif.2021.102008

[ref34] van Lieshout L. L. de Lange F. P. Cools R. (2021). Uncertainty increases curiosity, but decreases happiness. Sci. Rep. 11:14014. doi: 10.1038/s41598-021-93464-6 34234250 PMC8263743

[ref35] Wang Q. Zhang J. Xing C. (2021). Longshot or favorite: the ending effect in investment decisions. Front. Psychol. 12:708211. doi: 10.3389/fpsyg.2021.708211, PMID: 34795611 PMC8592974

[ref36] Wisniewski B. Zierer K. Hattie J. (2020). The power of feedback revisited: a meta-analysis of educational feedback research. Front. Psychol. 10:487662. doi: 10.3389/fpsyg.2019.03087 PMC6987456 32038429

[ref37] Xing C. Meng Y. Isaacowitz D. M. Wen Y. Lin Z. (2019). The ending effect in investment decisions: the motivational need for an emotionally rewarding ending. Personal. Soc. Psychol. Bull. 45, 510–527. doi: 10.1177/0146167218788829, PMID: 30145945

[ref38] Zhao Y. Huang Z. Wu Y. Peng K. (2023). Autonomy matters: influences of causality orientations on Chinese adolescents’ growth mindset. J. Pac. Rim Psychol. 17:e25. doi: 10.1177/18344909231157466

[ref39] Zhou J. (1998). Feedback valence, feedback style, task autonomy, and achievement orientation: interactive effects on creative performance. J. Appl. Psychol. 83, 261–276. doi: 10.1037/0021-9010.83.2.261

