# Supplementary material for: The impact of time-limited context and feedback methods on epistemic curiosity
Source: Front Psychol. 2025 Nov 17;16:1717846. doi: 10.3389/fpsyg.2025.1717846 (PMC12665758; doi:10.3389/fpsyg.2025.1717846)
Supplement: Supplementary file 1 [file Table_1.DOCX]

Supplementary Material

# Supplementary Table

**Supplementary Table 1.** Cognitive curiosity elicitation materials used in the study

| Question | Answer |
| --- | --- |
| In which ancient city were the "Hanging Gardens" located? | Babylon |
| Who was the first ruler of the Holy Roman Empire? | Charlemagne |
| Which country is the world's largest producer of olive oil? | Spain |
| Which land mammal has the highest blood pressure? | Giraffe |
| Which country has 73% of its area covered by forests? | Finland |
| What was the name of the largest German battleship sunk in WWII? | Bismarck |
| Which country was the first to grant women the right to vote? | New Zealand |
| What is the world's largest freshwater lake? | Lake Superior |
| Who was the first to use "V" as a symbol for victory? | Winston Churchill |
| In which country is Angel Falls, the world's tallest waterfall? | Venezuela |
| What did the "D" in DC Comics originally stand for? | Detective |
| What is the name of the mountain range that separates Asia and Europe? | Ural Mountains |
| What is the longest river in Asia? | Yangtze River |
| Who was the first FIFA World Cup champion? | Uruguay |
| What is the largest species of bear on Earth? | Polar Bear |
| In which city was the Titanic built? | Belfast |
| In which city is Michelangelo's statue of David located? | Florence |
| Which country has the highest population density? | Monaco |
| In emergencies, which fruit's liquid can be used as a plasma substitute? | Coconut |
| What is the brightest star in the sky? | Sirius |
| What was the final piece of music composed by Mozart? | Requiem |
| What is the most common blood type in humans? | Type O |
| Which famous Greek philosopher is said to have been Alexander the Great's tutor? | Aristotle |
| What is the world's largest island? | Greenland |
| Who composed the opera "Don Giovanni"? | Mozart |
| On which vegetable did ancient Egyptians place their right hand when taking an oath? | Onion |
| Where is the Earth's largest volcano located? | Hawaii |
| What color flag was originally used to indicate disease on a ship? | Yellow |
| Which language has the largest vocabulary? | English |
| Neptune's blue color comes from which gas? | Methane |
| What is the only food that does not spoil? | Honey |
| Who is the author of the narrative poem "Don Juan"? | Lord Byron |
| What is the fastest healing part of the human body? | Tongue |
| Who is known as the "Father of Geometry"? | Euclid |
| What is the national flower of Spain? | Carnation |
| What is the largest temple in Egypt? | Karnak Temple |
| What is the only tissue in the human body without a blood supply? | Cornea |
| What was used in thermometers before mercury? | Alcohol |
| What furry animal preys on cobras? | Mongoose |
| What was the name of Darwin's research ship? | HMS Beagle |
